# The impact of bariatric and metabolic surgery on the morbidity and mortality of patients infected during the COVID-19 pandemic: a retrospective cohort study

**DOI:** 10.1590/1516-3180.2021.0952.R2.11052022

**Published:** 2022-08-29

**Authors:** Luiz Henrique Sala de Melo Costa, Luiz Filipe Sala de Melo Costa, Gabriela Rezende Kachan, João Kleber de Almeida Gentile, Raul Andrade Mendonça, Marcela Ralin de Carvalho Deda Costa, Jurandir Marcondes Ribas

**Affiliations:** I MD. Physician and General Surgeon, Postgraduate Program in Digestive Tract Surgery, Colégio Brasileiro de Cirurgia Digestiva (CBCD), Aracaju (SE), Brazil; II Undergraduate Student of Medical Sciences, Faculdade de Medicina-Universidade Cidade de São Paulo (FM-UNICID), São Paulo (SP), Brazil.; III MD. Physician, Department of Medicine, Faculdade de Medicina-Universidade Cidade de São Paulo (FM-UNICID), São Paulo (SP), Brazil.; IV MD. Gastrosurgeon, General Surgeon, Doctoral Student, and Assistant Professor, Department of Surgery, Faculdade de Medicina-Universidade Cidade de São Paulo (FM-UNICID), São Paulo (SP), Brazil.; V MD. Gastrosurgeon and General Surgeon, Department of Urgency of Hospital de Urgências de Aracajú, Aracaju (SE), Brazil.; VI PhD. Professor, Department of Physiotherapy, Universidade Federal de Sergipe (UFS), Lagarto (SE), Brazil.; VII PhD. Physician and General Surgeon, Department of Digestive Surgery, Universidade Federal do Paraná (UFRP), Curitina (PR), Brazil.

**Keywords:** Bariatric surgery, Obesity, COVID-19, Body mass index, Comorbidities, Protective factor, Hospitalization rate, Infection rate

## Abstract

**BACKGROUND:**

Since the impact of the coronavirus disease 2019 (COVID-19) pandemic in March 2020, several studies have shown a strong relationship between obesity and severe cases of COVID-19. It is imperative to assess whether bariatric surgery exerts a protective effect in such cases.

**OBJECTIVE:**

This study aimed to assess the impact of bariatric surgery on the morbidity and mortality in obese patients during the COVID-19 pandemic. A comprehensive search was performed using the PubMed and Cochrane Library databases.

**DESIGN AND SETTING:**

Retrospective cohort studies conducted in the Faculdade de Medicina da Universidade Cidade de São Paulo, São Paulo (SP), Brazil.

**METHODS:**

The search comprised the following descriptors: “bariatric, surgery, COVID-19”. Current retrospective cohort studies that examined the influence of bariatric surgery on the morbidity and mortality of obese patients during the COVID-19 pandemic were considered eligible.

**RESULTS:**

After removing duplicates, 184 studies were obtained from the databases. Of these, 181 were excluded from the analysis as they did not meet the eligibility criteria. Patients undergoing postoperative follow-up of bariatric surgery had a similar probability of SARS-CoV-2 infection compared to the general population, and persistent comorbidities were associated with an increased risk and severity of infection.

**CONCLUSION:**

Bariatric surgery has a protective effect against severe COVID-19 in the obese population, bringing the prevalence of severe disease cases to levels equivalent to those of the nonobese general population, with a positive impact on morbidity and mortality.

## INTRODUCTION

In March 2020, the World Health Organization (WHO) declared the coronavirus disease 2019 (COVID-19) a pandemic. Since then, the impact of this infection on the public and private health systems of many countries has become evident.^1^ The overcrowding of intensive care beds has led to the cancellation of elective surgeries, as there has been an increasing demand for professionals and and resources to treat infected patients.^2,3^ In this context, Hussain et al.^4^ presented a flowchart scaling priority among candidates for elective and revision procedures during the pandemic. Patients with severe obesity, comorbidities, or surgical complications should be prioritized when performing procedures. Outpatient activities began to be performed through tele-medicine, and only urgent procedures such as early and late surgical complications remained in the usual routine.

Studies indicate obesity as an isolated risk factor for severe cases of COVID-19.^4–6^ In addition, biochemical and endocrine factors related to obesity, such as type 2 diabetes and insulin resistance, are worse prognostic factors in infected patients.^7,8^ Therefore, it has become imperative to evaluate whether bariatric surgery exerts a protective effect against severe covid-19 conditions. Retrospective studies have evaluated outcomes in patients with previous bariatric surgery infected with severe acute respiratory syndrome coronavirus 2 (SARS-CoV-2) regarding the severity of the disease, need for intensive care and impact on mortality.^6,9,10^ However, there remains a lack of controlled clinical trials or other prospective studies evaluating such parameters.

## OBJECTIVE

The present study aimed to evaluate, through a literature review, the impact of bariatric surgery on the morbidity and mortality of obese patients during the COVID-19 pandemic in reference centers inside and outside Brazil.

## METHODS

### Data sources and surveys

A comprehensive search was conducted using the PubMed and Cochrane Library databases. The search strategies comprised the following descriptors: “bariatric, surgery, COVID-19”. These have been adapted for use in various databases. The access routes to the descriptions of the studies used in this article are presented in Table 1.

**Table 1:** Comprehensive search strategy for research on bariatric and metabolic surgery during the coronavirus-2019 pandemic using harvesting information retrieval framework

Author (year)	Date searched	Article title	Journal	Search terms	Databases
Aminian et al.^9^ (2020)	September 15, 2021	Association of prior metabolic and bariatric surgery with severity of coronavirus disease 2019 (COVID-19) in patients with obesity	Official Journal of the American Society for Bariatric Surgery	Bariatric surgery; Obesity; COVID-19; Body mass index	PubMed
Bel Lassen et al.^10^ (2021)	September 15, 2021	COVID-19 and its Severity in Bariatric Surgery-Operated Patients	Obesity (Silver Spring)	Bariatric surgery; Obesity; COVID-19; Body mass index	PubMed
Uccelli et al.^6^ (2020)	September 15, 2021	COVID-19 and Obesity: Is Bariatric Surgery Protective? Retrospective Analysis on 2,145 Patients Undergone Bariatric-Metabolic Surgery from High Volume Center in Italy (Lombardy)	Obesity Surgery	Bariatric surgery; Obesity; COVID-19; Body mass index;	PubMed

Current retrospective cohort studies that examined the influence of bariatric surgery on the morbidity and mortality of obese patients during the COVID-19 pandemic were eligible for this review without restrictions on dates and languages.

Further inclusion criteria included studies that evaluated adult patients over 18 and under 65 years of age, obese patients who underwent bariatric surgery, and those infected by SARS-CoV-2, in reference centers inside and outside Brazil.

Studies with patients outside the age group of 18 to 65 years, those that did not deal with bariatric surgery, and those performed outside the pandemic period were excluded.

### Data extraction

Data extraction was performed using a standardized data extraction form. The data extracted from all studies included study details, demographic data of participants, and available information on the interventions used.

## RESULTS

### Search results

**Figure 1:**
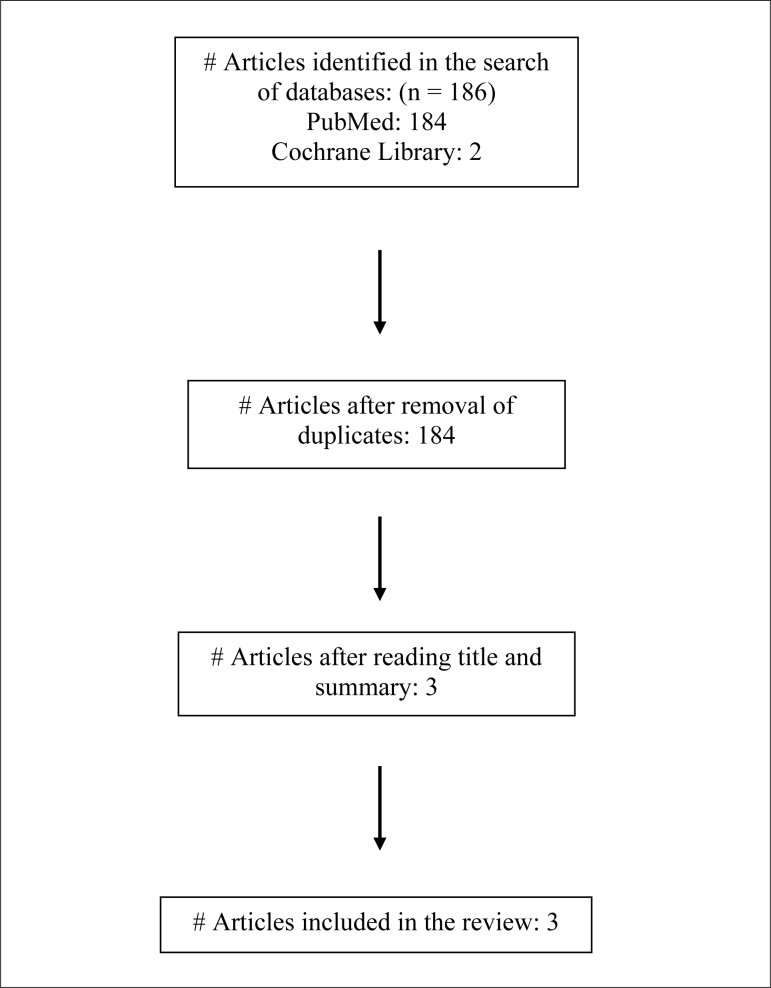
Flow diagram of the results.

A total of 186 studies were obtained from the surveyed databases. After removing duplicates, 184 studies were retained for the analysis. Of these, 181 were excluded after analyzing titles, abstracts, and full texts because they did not meet the eligibility criteria. Only three studies were included in this review (Figure 1). The characterization of the participants included in the studies is shown in Table 2.

**Table 2:** Characterization of the participants included in the study

Study	n	Average age (years)	Sex	Diagnosis
Aminian et al.^9^	4,365	46	Male and female	Obesity
Bel Lassen et al.^10^	738	50	Male and female	Obesity
Uccelli et al.^6^	2,145	44	Male and female	Obesity

A description of studies evaluating the impact of bariatric surgery on the morbidity and mortality of obese patients during the COVID-19 pandemic is shown in Table 3.

**Table 3:** Description of studies evaluating the impact of bariatric surgery on morbidity and mortality of obese patients during the coronavirus disease 2019 (COVID-19) pandemic

Study	Aminian et al.^9^	Bel Lassen et al.^10^	Uccelli et al.^6^
**Participants**	n = 363 tested positive for COVID-19 Group with previous surgery: 33; Group of non-operated: 330	n = 738; All underwent bariatric surgery Group “probably infected”: 62; Group “probably not infected”: 676	n = 2,145; All underwent bariatric surgery
**Goals**	Investigate the relationship between previous metabolic surgery and the severity of COVID-19 in patients with severe obesity.	Estimate the prevalence of COVID-19 and evaluate factors associated with the incidence and severity of the disease in patients who underwent bariatric surgery.	Investigate the incidence of SARS-CoV-2 infection and its severity in patients who underwent bariatric surgery.
**Collection procedures**	A search was performed in medical records of the institution that conducted the study for patients who tested positive in RT-PCR for COVID-19, evaluating the rate and time of hospitalization, need for ICU, mechanical ventilation, dialysis, and mortality in patients who tested positive in RT-PCR for COVID-19, evaluating the rate and time of hospitalization, need for ICU, mechanical ventilation, dialysis and mortality.	A standardized questionnaire was conducted through telephone calls in which probable symptoms of COVID-19 were questioned, such as anosmia, fever, rhinorrhea, odynophagia, or patients who tested positive for the disease. In addition, a medical record search was performed for anthropometric and laboratory data before and after the patients.	A questionnaire was sent to patients previously submitted to bariatric surgery in which age, gender, BMI, origin, comorbidities, and type of surgery were questioned, and they were asked about the main symptoms of COVID-19, and occurrence of hospitalization and ICU admission.
**Main findings**	The mean preoperative BMI in the group with previous surgery was 49.1 ± 8.8kg/m^2^, decreasing to 37.2 ± 7.1 kg/m^2^ at the time of testing for COVID-19. The mean BMI of the non-operated group was 46.7 ± 6.4 kg/m^2^. Six patients (18.2%) from the group submitted to surgery, and 139 patients (42.1%) from the non-operated group were admitted to the hospital (P = 0.013). 43 patients (13%) from the non-operated group required ICU admission (P = 0.021). 22 patients (6.7%) required mechanical ventilation. Five patients (1.5%) underwent dialysis. Eight patients (2.4%) died. In the group with previous surgery, none of these four outcomes were identified.	Patients had a mean age of 50 ± 12.3 years, with most being female (78.3%) and 44% having type 2 diabetes before surgery. The most used surgical technique was gastric bypass (54.4%), followed by sleeve gastrectomy (45.0%). The mean postoperative time at collection was 3.7 ± 2.7 years. There was no difference in the surgical technique outcomes between the groups. The mean postoperative time was significantly longer in the “probably infected” group, with a considerably higher proportion of persistently diabetic patients than in the “probably not infected” group.	All patients underwent elective bariatric surgery. The mean preoperative BMI was 44 ± 6.8 kg/m^2^ with a reduction to 29.3 ± 5.5 kg/m^2^ in the postoperative period. The main technique used was laparoscopic sleeve gastrectomy (82.4%). The reduction in the number of comorbidities was almost entirely statistically significant. A total of 181 patients (8.4%) reported at least one symptom related to COVID-19. Nevertheless, only 26 cases (1.2%) were tested, and only 13 individuals (0.6%) tested positive. Six patients (0.3%) were admitted to hospital units; two patients (0.1%) required ICU with mechanical ventilation. The mean length of hospital stay was 23 ± 13 days.
**Conclusions**	The study identified that previous bariatric surgery is associated with lower hospitalization rates and the need for ICU for patients infected with SARS-CoV-2.	Patients under postoperative follow-up of bariatric surgery presented a probability of SARS-CoV-2infection similar to that of the general population. The persistence of type 2 diabetes and the presence of lower BMI are associated with increased risk and severity of SARS-CoV-2 infection.	Because the rate of hospitalization and need for ICU of the patients evaluated was equivalent to those of the general nonobese population, the study concludes that bariatric surgery can be considered a protective factor for severe acute respiratory syndrome caused by SARS-CoV-2 infection

RT-PCR = reverse transcription polymerase chain reaction; ICU = intensive care unit; BMI = body mass index; SARS-CoV-2 = severe acute respiratory syndrome-coronavirus 2.

## DISCUSSION

Studies indicate obesity as an isolated risk factor for severe cases of COVID-19.^4–6^ In addition, biochemical and endocrine factors related to obesity, such as type 2 diabetes and insulin resistance, are associated with a worse prognosis in infected patients.^7,8,11^ In this context, the publications evaluated in this study explore bariatric surgery as an intervention capable of serving as a protective factor against severe cases of COVID-19.^6,9,10^ There is great heterogeneity between the methodology of the studies since the situation of social isolation itself made it impossible to conduct controlled clinical trials.

The publication by Uccelli et al.,^6^ whose data collection was carried out from March to May 2020, presented many participants from several different areas of Italy, which allowed a global analysis of the involved population. However, there was a population bias as only patients who had already undergone surgery answered the questionnaire, and there was no control group of non-surgical patients. There was also a low testing rate with reverse transcription polymerase chain reaction (RT-PCR) (1.2%), which may have underestimated the number of infected patients. Moreover, as the questionnaire was self-applicable online, seeking the most common symptoms of COVID-19, there was bias in the collection not being performed by an examiner trained to perform the necessary anamnesis.

The study conducted by Aminian et al.,^9^ whose data collection was carried out between March and July 2020, analyzed patients who tested positive for COVID-19 through RT-PCR and anthropometric data extracted from the institution's medical records confirmed the reliability of the research. However, the major limitation of this study was the small number of patients with a history of previous bariatric surgery, which resulted in a longer confidence interval and may have influenced the statistical analysis of the results. Moreover, as only six operated patients were hospitalized for COVID-19, laboratory, radiological, and oxygenation data were unavailable for most patients in this group; therefore, they were not included in the statistical analysis.

Bel Lassen et al.^10^ performed data collection between March and May 2020. Similar to the study by Aminian et al.,^9^ this study used anthropometric data collected from medical records with good reliability. Additionally, a large number of participants were included in the study. However, the postoperative time among the patients was extremely heterogeneous, with an interval of up to 16 years. This introduced a population bias that may have interfered with the results. Similar to the study by Uccelli et al.,^6^ a self-administered questionnaire was made available, which may have been subject to different interpretations by individuals regarding the symptoms of COVID-19.

Despite the heterogeneity in the methodology employed by the different authors and the complicating factors between data collection and statistical analysis of results, the three publications concluded that the prevalence of severe COVID-19 conditions in patients in the postoperative period of bariatric and metabolic surgery does not differ from the prevalence in the general nonobese population. From the perspective of countries' health systems that have managed COVID-19 in the long term, it is necessary to develop controlled clinical trials with a good methodology to assess whether such results are reproducible and whether there are other clinical implications in carrying out such procedures.

## CONCLUSION

Based on the results of the analyzed studies, even with the reservations described regarding the methodological limitations employed, it can be concluded that bariatric surgery exerts a protective effect against severe cases of COVID-19 in the obese population, with a positive impact on morbidity and mortality.
